# The American and Colonial Journals: Midwifery

**Published:** 1839-04

**Authors:** 


### MIDWIFERY.

### *Obstetrical Enormity.*

[The following case is related in a recent American journal, under the title of "Ramping Surgery!" We should have thought it fictitious, but for the narrator's giving his name and place of abode, viz. P. H. Hush, Oswego, N. Y.]

The woman was of middle age, good constitution, regularly formed. She was taken with labour for the fifth time, and after a lapse of several hours a seat was prepared on the side of the bed, on which she was placed. The husband, being seated, on examination found the right hand presenting. He made forcible extension on this, but could not succeed. The pains becoming insufferable, he noosed a piece of a bed-cord round the wrist of the child, and placing his foot against the bedstead and fundament of the patient, straightened back, with "*might and main,*" till the arm gave way, letting the operator, with his chair, over upon the floor, the arm flying to the back side of the room. This rather damped his zeal, and he started after a regular accoucheur; but just as the latter arrived, nature had expelled the fœtus without aid, by producing a spontaneous evolution, and giving the head a natural position. The child appeared large and sprightly. The father was directed to send immediately for a surgeon, to see what was to be done. He deferred eight hours (probably in hopes that death would aid in cloaking his infamy), at which time he called on me. I found the arm horribly mutilated; the humerus had given way one inch above the elbow-joint; the skin at the shoulders, the muscles, tendons, nerves, and blood-vessels, yielded at their weaker points; some oozing of blood. I amputated at the shoulder-joint, drew the integuments together, which united readily, and discharged him in fourteen days, well. He is now a healthy and sprightly child.

*Boston Med. and Surg. Journal. March* 21, 1838.

---

### III. THE BRITISH JOURNALS.

(FOR THE QUARTER ENDING FEBRUARY 28, 1839.)

